# Imaging-based observation of the pes region in the dromedary camel (*Camelus dromedarius*): computed tomography, 3D volume rendering, magnetic resonance imaging, and ultrasonography

**DOI:** 10.3389/fvets.2025.1558954

**Published:** 2025-04-24

**Authors:** Hazem Hamoda, Fahmy Gad Elsaid, Mamdouh Eldesoqui, Mai A. AL-Mosaibih, Eman Fayad, Mohamed K. Hussein, Mohamed A. Hamed, Foad Farrag, Mohamed Abumandour, Mustafa Shukry, Mohamed Abdelmegeid, Abanoub T. Yousef, Ahmed A. Elolimy

**Affiliations:** ^1^Department of Anatomy and Embryology, Faculty of Veterinary Medicine, Aswan University, Aswan, Egypt; ^2^Department of Biology, College of Science, King Khalid University, Abha, Saudi Arabia; ^3^Department of Basic Medical Sciences, College of Medicine, AlMaarefa University, Riyadh, Saudi Arabia; ^4^Department of Biological Sciences, College of Science, University of Jeddah, Jeddah, Saudi Arabia; ^5^Department of Biotechnology, College of Sciences, Taif University, Taif, Saudi Arabia; ^6^Department of Food Hygiene, Faculty of Veterinary Medicine, Aswan University, Aswan, Egypt; ^7^Department of Surgery, Anesthesiology, and Radiology, Faculty of Veterinary Medicine, Aswan University, Aswan, Egypt; ^8^Department of Anatomy and Embryology, Faculty of Veterinary Medicine, Kafrelsheikh University, Kafrelsheikh, Egypt; ^9^Department of Anatomy and Embryology, Faculty of Veterinary Medicine, Alexandria University, Alexandria, Egypt; ^10^Department of Physiology, Faculty of Veterinary Medicine, Kafrelsheikh University, Kafrelsheikh, Egypt; ^11^Veterinary Program, Faculty of Health Sciences, Higher Colleges of Technology, Sharjah, United Arab Emirates; ^12^Department of Integrative Agriculture, College of Agriculture and Veterinary Medicine, United Arab Emirates University, Abu Dhabi, United Arab Emirates

**Keywords:** *Camelus dromedaries*, computed tomography, ultrasonography, pes, MRI, 3D reconstruction render volume CT

## Abstract

**Introduction:**

Our investigations utilized computed tomography (CT), magnetic resonance imaging (MRI), and ultrasonography (US) to provide detailed anatomical information on the pes region of a one-humped camel, with a particular focus on 3D reconstruction volume-rendered CT (3DVR-CT).

**Methods:**

This research utilized 16 pes regions from adult male *Camelus dromedaries* aged 8–10 years.

**Results:**

A CT scan revealed a vertical bony septum that completely divides the internal medullary cavity of the fused large metatarsal bones along the fusion line, except for the distal one-fifth portion, where the septum was absent. MRI identified the ligaments of the fetlock joint, which include the axial and abaxial collateral ligaments, collateral sesamoidean ligaments, and palmar ligaments. US demonstrated that the deep digital flexor tendon (DDFT) had higher echogenicity than the superficial digital flexor tendon (SDFT) at all imaging levels, with the SDFT paratendon appearing hyperechoic, which differentiated it from both the SDFT and the suspensory ligament. Our application of CT and MRI imaging techniques revealed that the *Manica flexoria* and distal sesamoid bone were not observed. On MRI, the sole appeared as a layer of low signal intensity, while the digital cushion exhibited heterogeneous high signal intensity.

**Discussion:**

the evaluation of anatomical regions in *Camelus* dromedaries can now be performed using CT, MRI, and US with 3DVR-CT, greatly improving the diagnosis and treatment of various conditions and facilitating the interpretation of some clinical diseases in the pes region. These imaging modalities, particularly 3DVR-CT, serve as valuable tools for veterinary clinicians and researchers studying camel anatomy and pathology.

## 1 Introduction

Dromedary camels are renowned for their exceptional adaptability and ability to tolerate harsh conditions with limited sustenance, demonstrating their capacity to thrive in extreme environments such as deserts and icy regions (1–3). The *Camelidae* family comprises two genera: Old World camels (*Camelus*) and New World camels (Lama). Old World camels, such as the *dromedary*, inhabit hot deserts in Africa and Asia, while Bactrians reside in cold, arid grasslands. The Arabian one-humped camel, a member of the *Camelus dromedaries* species, lives in Egypt and can survive, reproduce, produce milk and meat, and work in extreme heat and arid conditions, even during droughts (4).

The pes region of a camel consists of the following bones: tarsal bones, metatarsal bones, and phalanges (5–7). The camel’s metatarsal skeleton was formed by the fused large third and fourth metatarsal bones. Lameness in camels’ distal limbs, often caused by injury or infection, requires early detection, treatment, regular veterinary check-ups, and proper hoof care for a successful recovery (7). Since lameness is a major problem affecting dairy animals and has a negative economic impact, injury sites must be quickly identified to facilitate a successful recovery (8). Lame Camels require a thorough examination of their causes and related variables to prevent and treat the condition, which can be complicated to identify and manage (9).

Computed tomography (CT) scans, while being a valuable imaging technique, are limited in veterinary medicine due to their exclusivity and the requirement for anesthesia (10, 11). CT is a valuable diagnostic tool because of its superior soft tissue contrast and the absence of overlapping structures compared to routine radiography (12–14). In the current era of clinical and radiological practice, the 3D reconstruction volume-rendered CT (3DVR-CT) imaging technique holds significant importance (11). 3DVR technology provides all the necessary data for a single study, thus reducing the need for multiple studies, which greatly enhances radiological procedures (15).

The non-invasive magnetic resonance imaging (MRI) technique provides accurate anatomical features of various body structures in both healthy and unhealthy individuals (14, 16). The application of this method has significantly improved diagnostic imaging techniques by offering a new perspective on different structures—particularly soft tissues (14). When selecting an MRI machine for veterinary care or research, factors such as examination requirements and cost must be considered. Low-field MRI is increasingly popular due to its accuracy and affordability in imaging distal limbs (17). MRI produces comprehensive images of the bones in the foot area, as well as their tendons, ligaments, and joints, aiding in the identification of fractures, sprains, arthritis, and defects that are not visible on X-rays (18). MRI is a vital diagnostic tool for veterinary radiologists and practitioners, necessitating a thorough understanding of foot anatomy to accurately interpret scans and diagnose conditions such as tumors, infections, and nerve injuries (14). The MRI technique has identified homogeneous, low-signal lateral digital extensor tendons and common digital extensor tendons, which are separated from adjacent fascia by low intermediate signal intensity (14).

Ultrasonography (US) is a beneficial imaging technique for identifying abnormalities in soft tissue, including tendonitis and tenosynovitis, which primarily depend on changes in size and echogenicity (19). It is essential to understand the typical echogenic appearance of the structures being examined to identify injury features, prevent misinterpretations, and avoid false positive diagnoses. According to Çlelimli et al. (13), the diagnostic US imaging technique is often considered the least expensive option for assessing soft tissue injuries.

The pes region of the one-humped camel has not been thoroughly examined using various imaging techniques, particularly MRI. Previously published data have focused solely on computed tomography combined with gross anatomical analysis (20, 21). Therefore, the current study aims to provide comprehensive anatomical information on the pes region of *Camelus dromedarius* through the use of CT, 3D volume reconstruction, MRI, and US. This study aimed to bridge the knowledge gap regarding the anatomy of the pes region in Arabian one-humped camels by utilizing multiple imaging techniques for a detailed and thorough examination.

## 2 Materials and methods

### 2.1 Animal’s collections

In this study, we examined 16 pes regions from eight adult male Arabian camels, each one-humped and aged 8 to 10 years. No musculoskeletal defects were found in any of the limbs. The pes regions were obtained from the Al-Shalaal slaughterhouse in the Aswan governorate, separated from the tarsometatarsal joint, and thoroughly examined for signs of trauma or disease before dissection. *Camelus dromedarius* were clinically assessed for lameness prior to slaughter through visual examination, observation of the animals in motion, and palpation of the tarsal, metatarsal, and pes regions. The pes regions were frozen at −20°C and examined within 2 h to prevent postmortem changes. The obtained pes regions were preserved in a normal saline solution for 2 h.

### 2.2 CT technique (*n* = 5)

**Figure 1:**
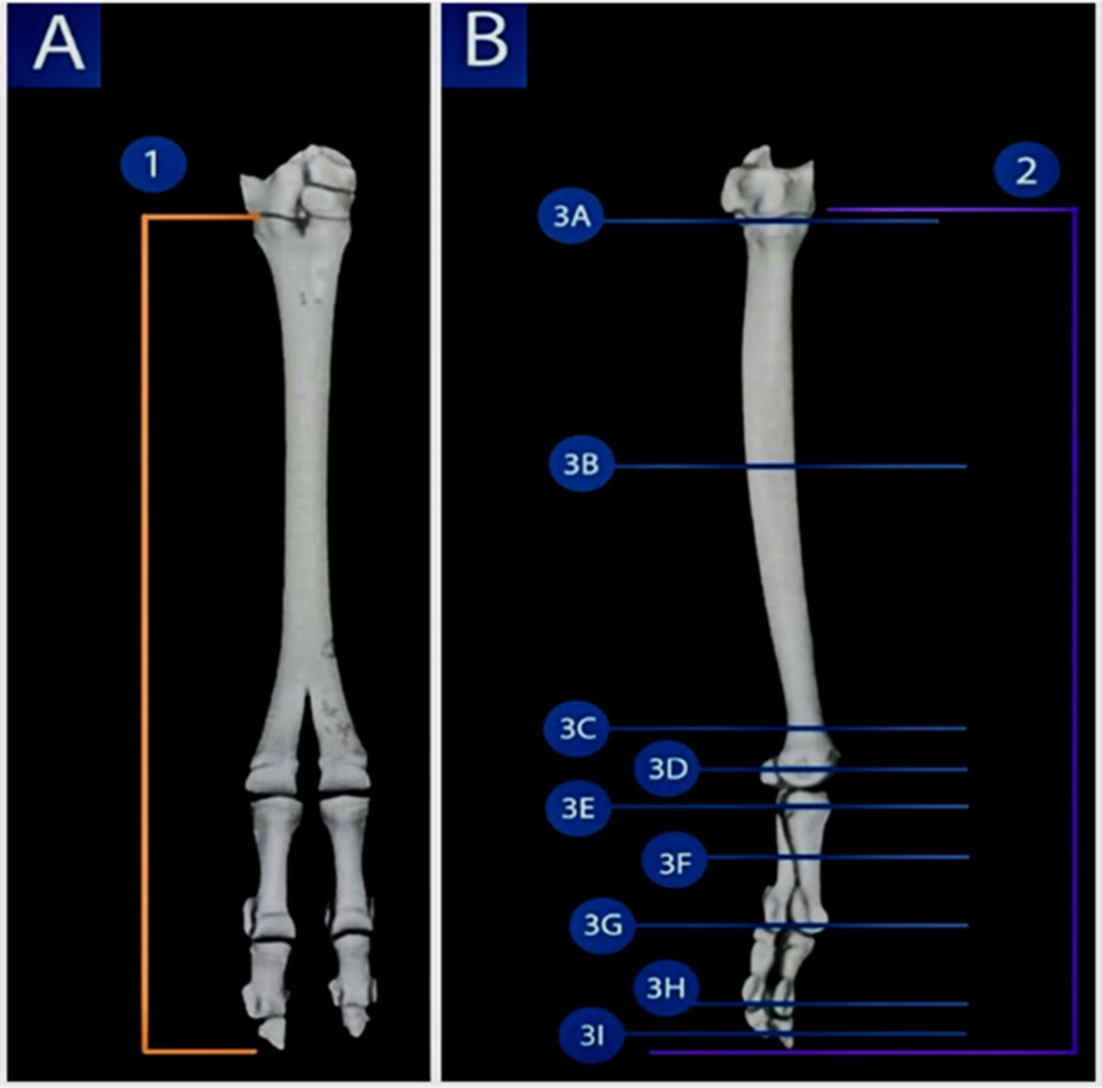
3D reconstruction render of a volume CT image of the pes region of the Arabian one-humped camel (*Camelus dromedarius*); the dorsal view shows the selected planes for the dorsal sections (view **A**), while the lateral view depicts the selected planes for the sagittal and transverse sections (view **B**), highlighting the imaging planes used in the present study for CT and MRI of the pes region.

The collected pes regions (*n* = 5) were rinsed under tap water to remove debris, then wrapped at the stump with a plastic sheath and stored at either 4°C (for CT scan) or 3°C (for 3DVR-CT) imaging techniques for 3D reconstructions from CT images. The pes regions were taken to the radiological CT center within 2 h following slaughter to undergo a subsequent CT scan using a CT scanner in helical scan mode, equipped with a 128-detector, multi-slice (16-slice) spiral Optima CT520 Series scanner (Siemens Healthiness CT device; Aquilion; Toshiba Medical Systems, Tokyo, Japan); scanning conditions: 120 kV, 100 mA, 2 s, window width: WW: 400; window level: WL 60 Hounsfield units, with a pitch of 0.625, a field of view of 45 cm, and a matrix size of 512 × 512 pixels set for soft window. A bone window was created by modifying the settings (window width: WW: 1,500; window level: WL: 300 Hounsfield units). A CT scan of 1 mm thickness was performed in contiguous transverse, dorsal, and sagittal images (Figure 1).

Nine CT transverse images of the pes region, including its digits, were processed, starting at the tibiotarsal joint proximally and continuing in sequence distally to the middle of the middle phalanx, just above the distal phalangeal joint. The transverse CT images were selected at the levels of the base, shaft, and distal extremity of the large metatarsal bone, the metatarsophalangeal joint, the proximal extremity of the proximal phalanx, the middle shaft of the proximal phalanx, the proximal interphalangeal (pastern) joint, the middle of the middle phalanx, and the distal interphalangeal (coffin) joint. The sagittal CT images were chosen from approximately 50 slices, each 1-mm thick, beginning from the medial aspect and moving toward the lateral aspect of the pes region. The coronal CT images were selected from 60 CT views, each with a thickness of 1 mm. Overall, the soft window images enabled the detection of the most clinically significant soft tissue structures, including the extensor tendons, superficial and deep digital flexor tendons, the digital cushion, collateral and sesamoidean ligaments, and the joint capsules of the digital joints.

Three-dimensional CT (3-DCT) imaging of the pes skeleton region was performed using the same CT system after adjusting its settings (window width, WW: 332; window level, WL: 287 Hounsfield units). For documentation, the scanned CT images were stored and printed with a CT digital printer, and the digital images were saved on a hard drive for offline analysis using Radiant DICOM Viewer software (version 2020.2.3; Medixant, Poznan, Poland) to reconstruct them in multiplanar reconstruction (MPR) and three dimensions (3D).

#### 2.2.1 Magnetic resonance imaging (MRI; *n* = 5)

The same previously examined pes regions (*n* = 5) were analyzed using consecutive MRI scans performed with a 1.5 Tesla magnetic field apparatus (Magneton Concerto, Siemens Germany) and a standard human body coil. A continuous series of transverse, dorsal, and sagittal scans were obtained from the pes regions (Figure 1). T1-weighted MRI images were acquired with the following parameters: Repetition time (TR) = 486 ms, echo time (TE) = 9.5 ms, 4 mm slice thickness, and 1 mm inter-slice spacing. Nine MRI transverse images of the pes region, including their digits, were taken. The transverse MRI images were selected at levels corresponding to the base of the large metatarsal bone, the shaft of the large metatarsal bone, the distal extremity of the large metatarsal bone, the metatarsophalangeal joint, the proximal extremity of the proximal phalanx, the middle of the body (shaft) of the proximal phalanx, the proximal interphalangeal joint, the middle of the middle phalanx, and the distal interphalangeal (coffin) joint.

#### 2.2.2 Ultrasonography (*n* = 5)

The collected pes regions (*n* = 5) were imaged using ultrasound within 2–4 h. For the ultrasound imaging procedure, the limbs were clipped, shaved, and washed with water, after which acoustic coupling gel was applied. An US examination was performed using a B-mode Mindray M5 portable device (Mindray Bio-medical Electronics Co., Ltd., Shenzhen, China) connected to a 5–8 MHz linear transducer, scanning both longitudinally (along the entire length) and transversely, as well as plantarly. The scans were done and printed.

## 3 Results

### 3.1 Computed tomography

#### 3.1.1 Transverse CT of the pes region

**Figure 2:**
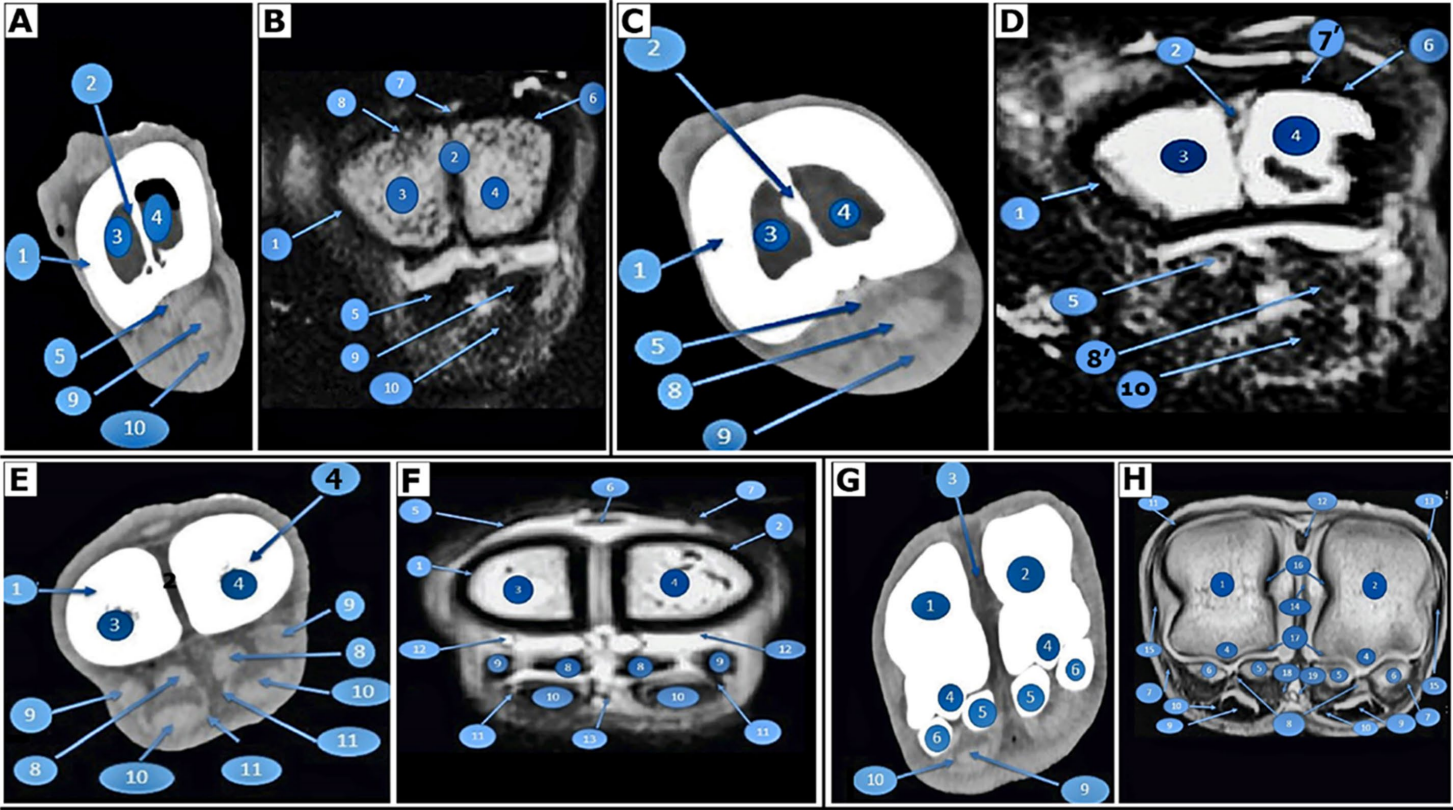
Transverse images of the right large metatarsal bone. At the base of the right large metatarsal bone, 3 cm distal to the tarsal articular surface of the pes region of *Camelus dromedarius*; CT (view **A**) and MRI (view **B**). In the middle of the right large metatarsal bone in the pes region of *Camelus dromedarius*, CT (view **C**) and MRI (view **D**) show the following structures: 1. fused third and fourth metatarsal bones; 2. bony septum; 3. medullary cavity of the fourth metatarsal bone; 4. medullary cavity of the third metatarsal bone; 5. interosseous muscle; 6. tendon of the long digital extensor muscle; 7. extensor digitorum brevis muscle; 7. tendon of the lateral digital extensor muscle; 8. tendon of the lateral digital extensor muscle; 8. tendon of the deep digital flexor muscle; 9. tendon of the deep digital flexor muscle; and 10. tendon of the superficial digital flexor muscle. At the distal extremity of the right large metatarsal bone, located 3 cm above the distal articular surface of the pes region of *Camelus dromedarius*, CT (view **E**) and MRI (view **F**) reveal the following structures: 1. fourth metatarsal bone; 2. third metatarsal bone; 3. tendon of the lateral digital extensor muscle; 4. lateral tendon of the long digital extensor muscle; 5. medial tendon of the long digital extensor muscle; 6. axial part of the interosseous muscle; 7. abaxial part of the interosseous muscle; 8. tendon of the deep digital flexor muscle; 9. tendon of the superficial digital flexor muscle; 10. plantar synovial pouch of the fetlock joint; and 11. common plantar digital artery. At the level of the proximal sesamoid bone or the right metatarsophalangeal (fetlock) joint in the pes region of *Camelus dromedarius*, CT (view **G**) and MRI (view **H**) reveal the following structures: 1. the distal end of the fourth metatarsal bone; 2. the distal end of the third metatarsal bone; 3. intertrochlear notch; 4. plantar sagittal ridge; 5. axial proximal sesamoid bones; 6. abaxial proximal sesamoid bones; 7. collateral sesamoidean ligament; 8. plantar ligament of the fetlock joint; 9. tendon of the deep digital flexor muscle (divided); 10. tendon of the superficial digital flexor muscle; 11. tendon of the lateral digital extensor muscle; 12. the lateral limb of the long digital extensor tendon; 13. the medial limb of the long digital extensor tendon; 14. axial collateral ligaments of the fetlock joint; 15. abaxial collateral ligaments of the fetlock joint; 16. dorsal synovial pouch of the fetlock joint; 17. plantar synovial pouch of the fetlock joint; 18. common plantar digital artery; and 19. common plantar digital vein.

Our findings were based on nine transverse CT images of the metatarsal bones and their digits. The fusion between the third and fourth metatarsal bones resulted in the formation of the metatarsus skeleton (Figure 1B1). This fusion was incomplete, as the bones separated at the distal one-fifth. The third and fourth metatarsal bones fused, and their proximal ends featured an internal vertical bony septum (Figure 1B2). To fully divide the medullary cavity of the fused bones’ proximal extremity, this septum was extended along the line of fusion between the two bones (Figures 1B3,4). A vertical bony septum was also visible internally in the shaft of the fused third and fourth metatarsal bones (Figure 2). This septum completely divided the medullary cavity (Figures 2C3,4). At the distal extremity (head), the two metatarsal bones were separated in the distal one-fifth due to the absence of the vertical bony septum (Figures 2E1,2).

On the dorsal aspect of the fused third and fourth metatarsal bones, the extensor tendons appeared on CT scans as a narrow, undifferentiated transverse strap; however, the outline of each tendon was not clearly defined. CT scans of the plantar aspect of the interosseous muscle (Figures 2A5,C5) showed the superficial digital flexor tendon (SDFT; Figures 2A10,C9,E11) and the deep digital flexor tendon (DDFT; Figures 2A9,C8,E10) located on the plantar side of the fused third and fourth metatarsal bones. At the distal end of the fused third and fourth metatarsal bones, the *interosseous medius muscle* (suspensory ligament) appeared as a hyperdense structure and could be clearly visualized as four oval structures representing axial and abaxial parts (Figures 2E8,9), in addition to the deep and SDFTs. Each hind limb contained two metatarsophalangeal (fetlock) joints, one for each digit. The articular cavity was a potential cavity, so it did not show in the CT images. The axial and abaxial proximal sesamoid bones of each metatarsophalangeal joint appeared in the CT images (Figures 2G5,6). The extensor structures were observed in the CT images only as a narrow, indistinguishable transverse strap on the dorsal surface of the proximal and middle phalanges.

**Figure 3:**
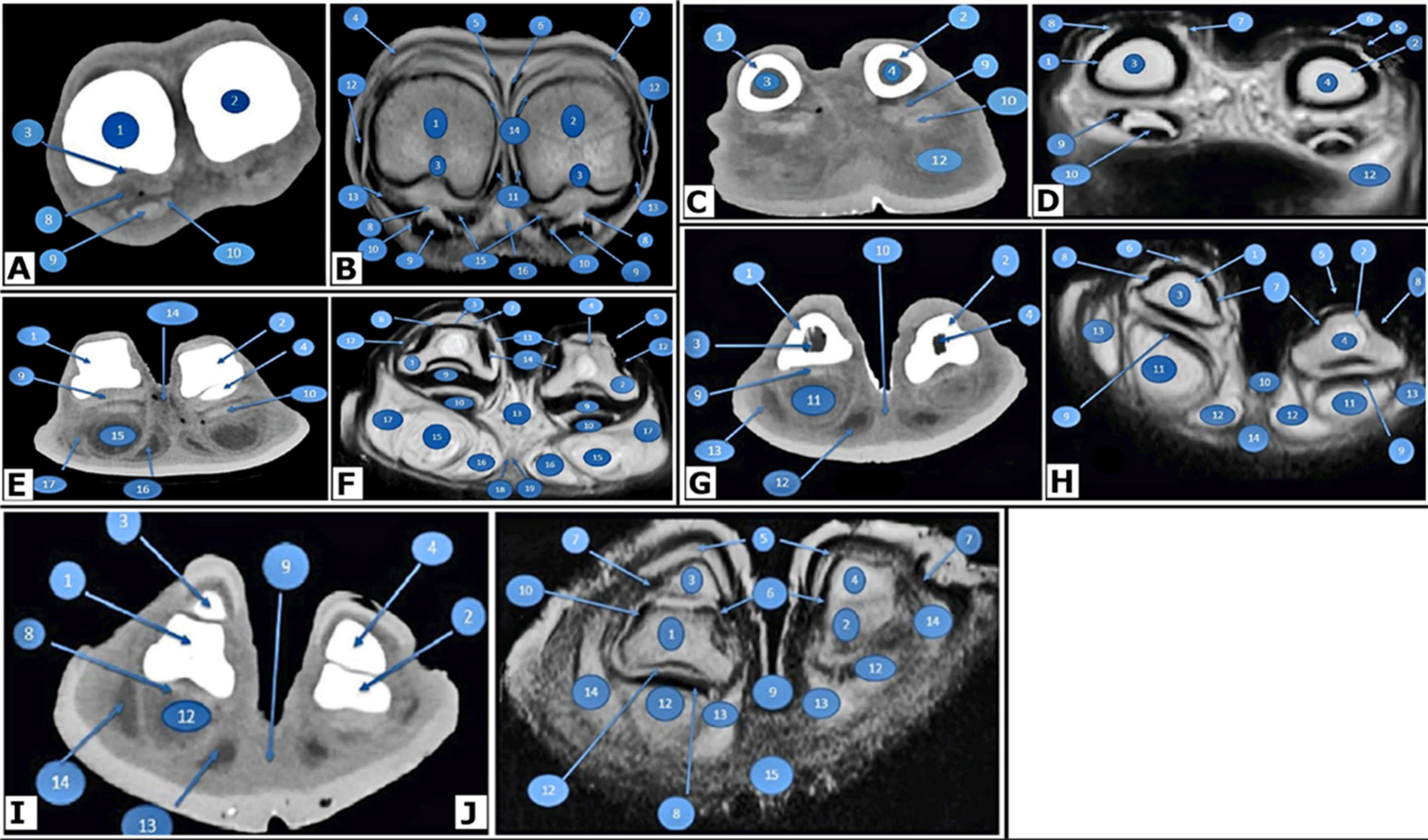
Transverse images of the right hind digits of *Camelus dromedarius.* At the proximal end of the proximal phalanx in the pes region, CT (view **A**) and MRI (view **B**) reveal the following structures: 1. the proximal end of the proximal phalanx of the fourth digit; 2. the proximal end of the proximal phalanx of the third digit; 3. trochlear fovea of the proximal phalanx; 4. tendon of the lateral digital extensor muscle; 5. lateral branch of the lateral tendon of the long digital extensor tendon; 6. medial branch of the long digital extensor tendon; 7. medial tendon of the common digital extensor tendon; 8. interosseous muscle; 9. tendon of the deep digital flexor muscle; 10. tendon of the superficial digital flexor muscle; 11. axial collateral ligaments of the fetlock joint; 12. abaxial collateral ligaments of the fetlock joint; 13. straight sesamoidean ligaments; 14. dorsal synovial pouch of the fetlock joint; 15. plantar synovial pouch of the fetlock joint; and 16. common plantar digital artery. Found in the middle (body or shaft) of the proximal phalanx in the pes region, CT (view **C**) and MRI (view **D**) reveal the following structures: 1. body of the proximal phalanx of the fourth digit; 2. body of the proximal phalanx of the third digit; 3. medullary cavity of the body of the proximal phalanx of the fourth digit; 4. medullary cavity of the body of the proximal phalanx of the third digit; 5. tendon of the lateral digital extensor muscle; 6. lateral branch of the lateral tendon of the long digital extensor; 7. medial branch of the lateral tendon of the long digital extensor; 8. medial tendon of the common digital extensor; 9. tendon of the superficial digital flexor muscle; 10. tendon of the deep digital flexor muscle; 11. plantar synovial pouch of the pastern joint; and 12. digital cushion. At the level of the proximal interphalangeal (pastern) joint in the pes region, CT (view **E**) and MRI (view **F**) reveal the following structures: 1. distal end of the proximal phalanx of the fourth digit; 2. proximal end of the middle phalanx of the fourth digit; 3. distal end of the proximal phalanx of the third digit; 4. proximal end of the middle phalanx of the third digit; 5. tendon of the lateral digital extensor muscle; 6. lateral branch of the lateral tendon of the long digital extensor tendon; 7. medial branch of the lateral tendon of the long digital extensor tendon; 8. medial tendon of the common digital extensor; 9. middle scutum; 10. tendons of the superficial and deep digital flexor muscles; 11. axial collateral ligaments of the pastern joint; 12. abaxial collateral ligaments of the pastern joint; 13. joint capsule of the pastern joint; 14. interdigital ligament; 15. middle digital cushion; 16. axial digital cushion; 17. abaxial digital cushion; 18. proper plantar digital artery; and 19. proper plantar digital vein. In the body of the middle phalanx of the pes region, CT (view **G**) and MRI (view **H**) reveal the following structures: 1. body of the middle phalanx of the fourth digit; 2. body of the middle phalanx of the third digit; 3. medullary cavity of the middle phalanx of the fourth digit; 4. medullary cavity of the middle phalanx of the third digit; 5. lateral branch of the long digital extensor tendon; 6. medial branch of the long digital extensor tendon; 7. axial dorsal ligaments of the coffin joint; 8. abaxial dorsal ligaments of the coffin joint; 9. tendon of the deep digital flexor muscle; 10. interdigital ligament; 11. middle digital cushion; 12. axial digital cushion; 13. abaxial digital cushion; and 14. sole. At the level of the distal interphalangeal (coffin) joint in the pes region, CT (view **I**) and MRI (view **J**) show the following structures: 1. distal end of the middle phalanx of the fourth digit; 2. distal end of the middle phalanx of the third digit; 3. distal phalanx of the fourth digit; 4. distal phalanx of the third digit; 5. nails; 6. axial collateral ligaments of the coffin joint; 7. abaxial collateral ligaments of the coffin joint; 8. tendon of the deep digital flexor muscle; 9. interdigital ligament; 10. joint capsule of the coffin joint; 11. plantar synovial pouch of the coffin joint; 12. middle digital cushion; 13. axial portion of the digital cushion; 14. abaxial cushion; 15. sole.

On the plantar surface of the digits, the DDFT and the SDFT appeared as rounded gray masses in the CT images, while their frameworks were indistinguishable. The SDFT (Figures 3A10,C9,E10) occupied a deeper position than the DDFT (Figures 3A9,C10,E10,G10), located just distal to the fetlock joint and anterior to its insertion at the base of the middle phalanx. The head of the proximal phalanx and the base of the middle phalanx articulated to form the proximal interphalangeal joint, also referred to as the pastern joint. The CT images did not reveal the articular cavity, as it is a potential cavity.

The medullary cavities of the proximal and middle phalanges exhibited low density and were clearly visible (Figures 3C3,4,G3,4,I3, 4). The plantar portion of the articular surface of the head of the proximal phalanx interacted with the articular surface of the middle scutum (Figure 3E9). The distal phalanx and the head of the middle phalanx articulated to form the distal interphalangeal (coffin) joint. The articular cavity was a prospective cavity; therefore, it did not appear in the CT images and was constrained plantarly by the digital cushions.

In CT scans, the digital cushions appeared clear and were divided into axial, middle, and abaxial (Figures 3A11,12,13,C12,E15,16). The third and fourth digits of the camel were joined by the interdigital ligament, which extended distally to the level of the coffin joint from the proximal interphalangeal joint (Figures 3E14,G10,I9).

#### 3.1.2 3DVR-CT scans of the pes region

**Figure 4:**
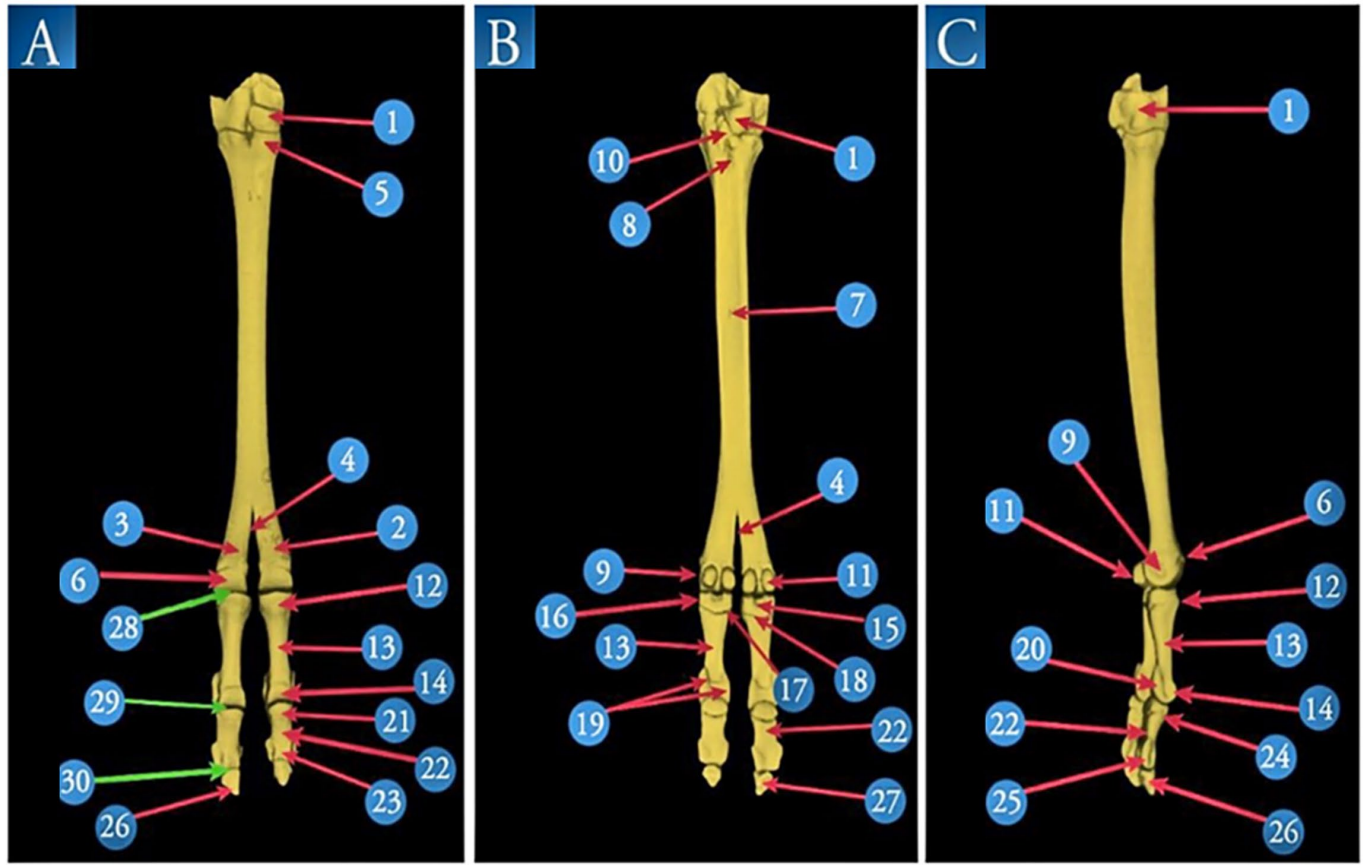
A 3D volume reconstruction of the CT scan of the right pes region of *Camelus dromedarius*, showing the following views: dorsal (view **A**), plantar (view **B**), and lateral (view **C**). It reveals the following structures: 1. tarsal bones; 2. third metatarsal bones; 3. fourth metatarsal bones; 4. intertrochlear notch; 5. tuberosity of the third metatarsal bone; 6. trochlea of the metatarsal articular surface; 7. two nutrient foramina; 8. proximal metatarsal canal; 9. tubercle and depression on the abaxial aspect of the metatarsal head; 10. pointed plantar process of the tarsal articular surface; 11. proximal sesamoid bones; 12. proximal extremity of the proximal phalanx; 13. shaft of the proximal phalanx; 14. distal extremity of the proximal phalanx; 15. sagittal notch; 16. rough depressed area on the abaxial aspect of the proximal phalanx; 17. tubercle and rough depressed area on the axial aspect of the base of the proximal phalanx; 18. triangular rough area; 19. axial and abaxial prominences; 20. depression for the abaxial collateral ligament of the proximal phalanx; 21. proximal extremity of the middle phalanx; 22. shaft of the middle phalanx; 23. distal extremity of the middle phalanx; 24. extensor process on the transverse ridge of the middle phalanx; 25. a depression for ligaments on each side (axially and abaxially) of the distal extremity of the middle phalanx; 26. margo dorsalis of the distal phalanx; 27. solar surface of the distal phalanx; 28. fetlock joint; 29. pastern joint; and 30. coffin joint.

In the dorsal view, the remaining distal row of tarsal bones appeared (Figure 4A1). The only remaining metatarsal bones were the third and fourth, which fused along their lengths except at the distal fifth, where they separated to form independent articulations with the corresponding digits (Figures 4A2,3). The distal extremity of the metatarsus was divided by the intertrochlear notch, which appeared inverted and v-shaped, creating two trochleae (Figure 4A4). Each trochlea was divided into two condyles, each smooth dorsally and separated by the intercondylar ridge; plantarly, it was divided into two areas: abaxial and axial (Figure 4A6). Its shaft is slender and has a more square shape. The tuberosity of the third metatarsal bone was located dorsally and proximally on the third metatarsal bone (Figure 4A5).

The proximal phalanx was the longest of the digit bones (Figures 4A12,13,14) and had a nearly semi-cylindrical shape. Its proximal extremity articulated with the distal extremity of the third and fourth metatarsal bones, forming the fetlock joint (Figure 4A28). The middle phalanx was approximately half the size of the proximal phalanx, compressed dorsoplantar and convex on its dorsal side (Figures 4A21,22,23). The pastern joint was created by the articulation between the distal extremity of the proximal phalanx and the proximal extremity of the middle phalanx (Figure 4A29). The distal phalanx had a wedge shape, with its apex directed dorsally. The Margodorsalis divided its dorsal surface into axial and abaxial parts (Figure 4A26). The coffin joint was formed by the distal extremity of the middle phalanx and the proximal extremity of the distal phalanx (Figure 4A30).

From the plantar view, the remaining distal row of tarsal bones appeared (Figure 4B1). The third and fourth metatarsal bones were fused. Two nutrient foramina were present along this surface, located above the middle of the shaft (Figure 4B7). The intertrochlear notch was visible at the distal extremity (Figure 4B4). The tarsal articular surface was characterized by a pointed process that occurred plantarly (Figure 4B10). Two proximal sesamoid bones articulated with the plantar surface of the distal extremity (head) of the third and fourth metatarsal bones (Figure 4B11). A tubercle and depression were noted on the abaxial aspect of the metatarsal head (Figure 4B9). The opening of the proximal metatarsal canal was small (Figure 4B8). The distal metatarsal canal was absent in the camel.

The articular surface of the proximal extremity of the proximal phalanx was flattened on the plantar side. It had two articular facets separated by an intermediate sagittal notch (Figure 4B15). A rough, depressed area appeared on the abaxial aspect of the proximal phalanx (Figure 4B16), while both a tubercle and a rough area were observed on the abaxial surface (Figure 4B17). The body had a roughly triangular region on the proximal one-third of its plantar surface (Figure 4B18). At its distal extremity, there were axial and abaxial prominences separated by a groove (Figure 4B19). The middle phalanx is flattened on the plantar side. The solar surface of the distal phalanx was also observed (Figure 4B27).

In the lateral view, the remaining distal row of tarsal bones was visible (Figure 4C1). A tubercle and a depression were present on the abaxial aspect of the metatarsal head (Figure 4C9). The trochlea of the metatarsal articular surface (Figure 4C6) and the two proximal sesamoid bones were also visible (Figure 4C11). The proximal articular surface of the proximal phalanx was slightly concave, with a depression noted for the abaxial collateral ligament (Figure 4C20). The middle phalanx had an extensor process that was evident on the transverse ridge of its proximal extremity (Figure 4C24). Depressions for the ligaments were observed on each side (axially and abaxially) of its distal extremity (Figure 4C25). The distal phalanx was also observed (Figure 4C26).

#### 3.1.3 Sagittal CT view of the pes region

**Figure 5:**
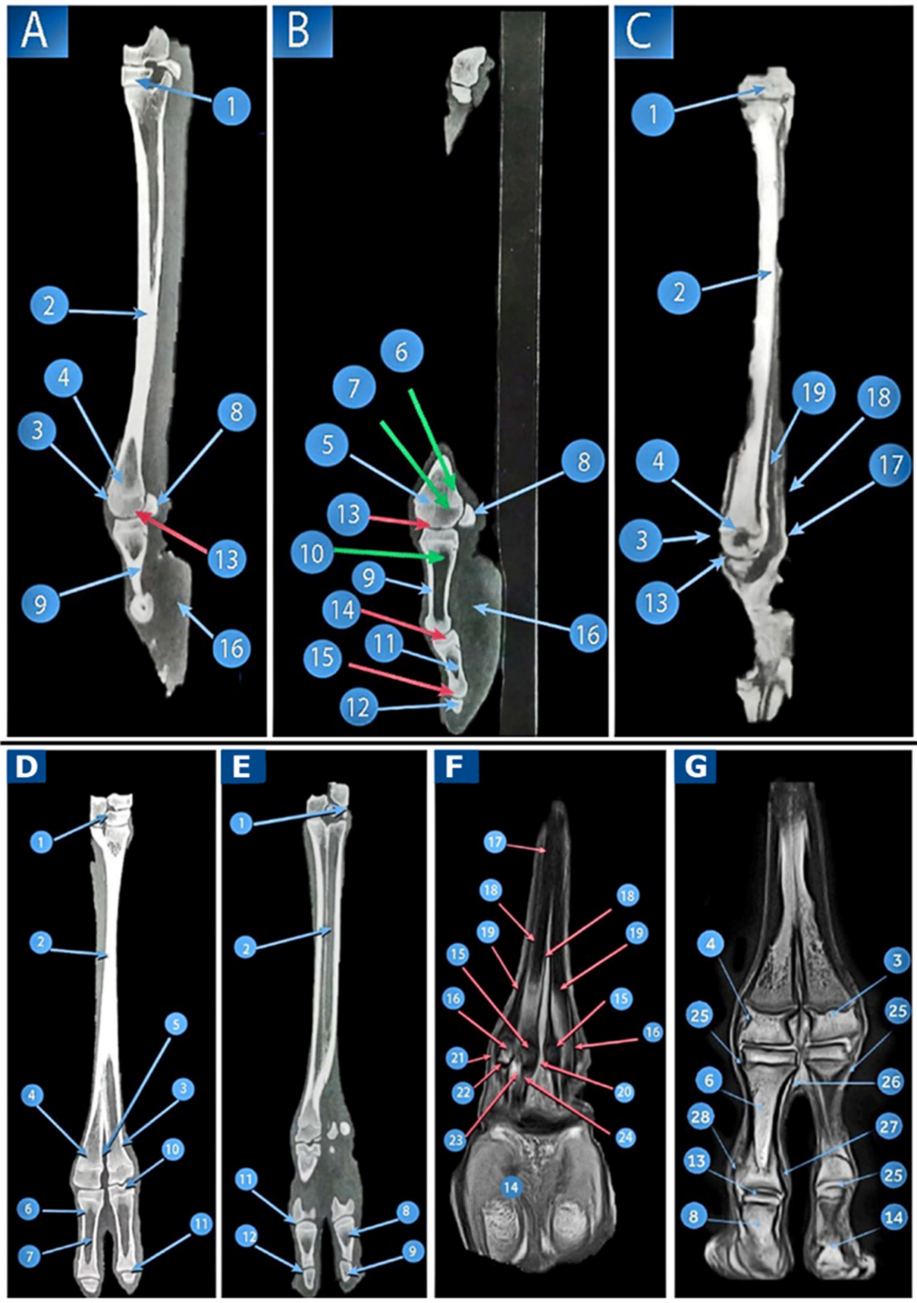
CT and MRI of the pes region of *Camelus dromedaries*. Sagittal images: CT of the pes region (Views **A,B**), CT of the distal end of the pes region (View **C**), and MRI of the pes region show the following structures: 1. tarsal bones; 2. large metatarsal bones; 3. trochlea of the metatarsal articular surface; 4. tubercle and depression on the abaxial aspect of the metatarsal head; 5. distal extremity of the metatarsal bones articulating with the third digit; 6. articular cartilage of the large metatarsal; 7. cancellous bone of the large metatarsal; 8. proximal sesamoid bones; 9. cortical bone of the proximal phalanx; 10. proximal phalanx; 11. middle phalanx; 12. distal phalanx; 13. fetlock joint; 14. pastern joint; 15. coffin joint; 16. Adipoelastic cushion; 17. SDFT; 18. deep digital flexor tendon; and 19. suspensory ligament. Dorsoplantar images, CT of the pes region (Views **D,E**), dorsal MRI at the level of the proximal sesamoid bone (View **F**), and dorsal MRI image at the distal end of the pes region (View **G**) reveal the following structures: 1. tarsal bone; 2. fused metatarsals; 3. distal extremities of the third metatarsal bones; 4. distal extremities of the fourth metatarsal bones; 5. intertrochlear notch; 6. proximal phalanx; 7. medullary cavity of the proximal phalanx; 8. middle phalanx; 9. distal phalanx; 10. fetlock joint; 11. pastern joint; 12. coffin joint; 13. articular cartilage; 14. adipoelastic digital cushion; 15. axial sesamoid bones; 16. abaxial sesamoid bones; 17. interosseous muscle; 18. axial part of the interosseous muscle; 19. abaxial part of the interosseous muscle; 20. axial collateral sesamoidean ligaments; 21. abaxial collateral sesamoidean ligaments; 22. straight sesamoidean ligaments; 23. deep digital flexor tendon; 24. SDFT; 25. axial collateral ligament of the fetlock joint; 26. abaxial collateral ligaments of the fetlock joint; 27. axial collateral ligament of the pastern joint; and 28. abaxial collateral ligaments of the pastern joint.

The remaining distal row of tarsal bones was observed (Figure 5A1). The large metatarsal bone was visible in the sagittal view (Figure 5A2). A tubercle and depression were noted on the abaxial aspect of the metatarsal head (Figure 5A4). The trochlea of the metatarsal articular surface (Figure 5A3) and the two proximal sesamoid bones (Figures 5A,B8) were also present.

The articular cartilage and cancellous bone of the large metatarsal bones were observed (Figures 5B6,7). At the extremities of the second and third phalanges, a moderate density of cancellous bone was noted. The cortical bone exhibited a high density, while the medullary cavities of the first and second phalanges displayed a low density (Figures 5B9,11). The distal phalanx was also examined. On the sagittal CT scans, the digital cushion pads showed low density in the middle and fairly moderate density around their capsules (Figures 5A,B16).

#### 3.1.4 Dorsopalmar view of the pes region

In the dorsal view, the distal row of remaining tarsal bones was visible (Figure 3A1). A fusion occurred between the third and fourth metatarsal bones (Figures 5D,E2). The two bones were fused, except at the distal fifth, where they diverged to form separate articulations with the corresponding digits. The distal extremity of the metacarpus was divided by an intertrochlear notch, which was observed to be inverted and V-shaped, creating two trochleae (Figure 5D5). Its shaft was more slender and square in diameter than that of the metacarpal bones.

At the extremities of the proximal and middle phalanges, the cancellous bone exhibited moderate density. Although the cortical bone showed high density, the medullary cavity of the proximal and intermediate phalanges displayed low density (Figures 5D7,8).

### 3.2 Magnetic resonance imaging

#### 3.2.1 Transverse magnetic resonance imaging of pes region

Nine MRI transverse images of the digits and metatarsus were used in our investigation. The vertical bony septum (Figure 2B2) was observed internally at the proximal extremity (base) of the fused third and fourth metatarsal bones (Figure 2B1). This septum extended along the fusion line between the two bones, completely dividing the medullary cavity at the proximal extremity of the fused bones (Figures 2B3,4). Extensor tendons, including the tendon of the lateral digital extensor muscle (Figure 2B8), the long digital extensor tendon (Figure 2B6), and the short digital extensor muscle (Figure 2B7) were identified. An internal vertical bony septum (Figure 2D2) was present in the shaft of the fused third and fourth metatarsal bones (Figure 2D1). The medullary cavity was completely divided by this septum (Figures 2D3,4). Tendons of the lateral digital extensor muscle (Figure 2D7) and the long digital extensor muscle (Figure 2D6) were also noted.

At the proximal and shaft of the fused bones, the flexor tendons were identified as *the interosseous medius muscle* (Figures 2B5, 3B5), along with the deep (Figures 2B9, 3B8) and SDFTs (Figures 2B10,D9), which displayed a black color and a hyperintense signal. The vertical bony septum was absent at the distal extremities (head) of the fused third and fourth metatarsal bones (Figures 2D1,2), resulting in the separation of the two metatarsal bones at the distal fifth. The lateral and long digital extensor tendons were visible (Figures 2D5,6,7) and appeared as homogeneous low-signal structures. The margins of these tendons exhibited an intermediate signal intensity and were observed as three narrow strips from the dorsal view of the distal extremity of the large metatarsal bone. The *interosseous medius muscle* (suspensory ligament) was clearly identified as four oval structures on the plantar aspect of the large metatarsal bone, represented in the axial and abaxial sections (Figures 2D8,9).

Each hind limb contained two metatarsophalangeal (fetlock) joints, one for each digit. Since the articular cavity was a potential cavity, the MRI images did not display it. A plantar ligament (Figure 2H8) was used to identify and connect the axial and abaxial proximal sesamoid bones (Figures 2H5,6) of each metatarsophalangeal joint. A collateral sesamoidean ligament connected each abaxial proximal sesamoid bone to the corresponding (medial or lateral) part of the distal extremity of the fused thi^rd^ and four^th^ metatarsal bones (Figure 2H7). Additionally, the lateral and long digital extensor tendons, among other soft tissue components, were observed (Figures 2H11,12,13).

The fetlock joint capsules were clearly visible as low signal intensities, with a thin line of intermediate signal intensity along their boundaries (Figures 2H16,17, 3B14,15). However, the ligaments of this joint were well-defined and clearly outlined, displaying heterogeneous intermediate signal intensities. The acquired MRI images enabled clear identification of the axial and abaxial collateral ligaments, collateral sesamoidean ligaments, and palmar ligaments (Figures 2H7,8,14,15). However, the cruciate and short sesamoidean ligaments were not visible.

On the dorsum of the proximal and middle phalanges, the extensors include the tendon of *M. extensor digitorum lateralis* (Figures 3B4,D5,F5). The tendon of *M. extensor digitorum longus* (Figures 3B5,6,7,D6,7,8,F6,7,8) is divided into two branches, which appear as a small oval structure on the dorsomedial aspect of a digit, resulting in the extensors manifesting as four distinct structures. These tendons are observed as homogeneous low-signal intensities, with their margins exhibiting a low intermediate signal intensity.

The digital flexor tendon was found to have homogenous low-signal intensity structures surrounded by a low-signal intensity digital tendon sheath (Figures 2F10,H9, 3B9). The DDFT had an oval shape and was surrounded by the SDFT (Figures 2F11,H10, 3B10) until it reached the level of the proximal extremity of the proximal phalanx. In the middle of the first phalanx, the SDFT (Figure 3D9) appeared deeper compared to the DDFT (Figure 3D10). Before its insertion at the proximal extremity of the middle phalanx, the DDFT, located plantar to the middle scutum (Figure 3F9), presented as a semi-circular structure (Figure 3F10). Conversely, distal to the pastern joint, this tendon became flattened (Figure 3H9).

The distal end of the proximal phalanx and the proximal end of the middle phalanx articulate to form the proximal interphalangeal joint, also known as the pastern joint. The medullary cavity of the second phalanx exhibited a high signal intensity (Figures 3H3,4). The distal end of the middle phalanx and the distal phalanx articulate to create the distal interphalangeal (coffin) joint. The articular cavity was not visible in the MRI images because it was a potential cavity.

The joint capsules of the pastern and coffin joints were observed to have a low signal intensity, while their margins were drawn as thin lines of intermediate signal intensity in these images (Figures 3D11,F11,J10,11). The ligaments of these joints displayed intermediate signal intensity. The ligaments that were visibly outlined on the MR images included the collateral ligaments of the pastern joint (Figures 3F11,12) as well as the collateral and dorsal ligaments of the coffin joint (Figures 3H7,8,J6,7). The plantar ligaments of the pastern joint and the ligaments of the navicular cartilage were not clearly visible.

The interdigital ligament links the third and fourth digits at the level of the proximal interphalangeal joint and continues to the coffin joint level (Figures 3F14,H10,J9). The sole of the footpad was observed as a layer of low signal intensity between two lines of high signal intensity (Figures 3H14,J15). The nails exhibited heterogeneous high signal intensity (Figure 3J5). The digital cushion also displayed heterogeneous high signal intensity (Figures 3D12,F15,16,17,H11,12,13,J12,13,14), divided into axial, middle, and abaxial sections. The signal intensity of blood vessels (Figures 2F13,H18,19, 3H16) appeared as a hyper-intense signal, appearing dark.

#### 3.2.2 Sagittal (MRI) view of the pes region

The remaining distal row of tarsal bones was visible in this view (Figure 5C1). The large metatarsal bone was also observed (Figure 5C2). The abaxial face of the metatarsal head has a depression and a tubercle (Figure 5C4). The trochlea of the metatarsal articular surface was also observed (Figure 5C3). The fetlock joint (Figure 5C13) was noticed; the suspensory ligament (Figure 5C19) was noticeable deep within the DDFT (Figure 5C18), and the SDFT (Figure 5C17) was also visible.

#### 3.2.3 Dorsopalmar (MRI) view of pes region

The distal extremities of the third and fourth metatarsal bones were examined in this view (Figures 5G3,4). The most accurate location to identify the interosseous medius muscle and its branches was at the level of the proximal sesamoid bones (Figures 5F17,18,19). Both the axial and abaxial proximal sesamoid bones were observed (Figures 5F15,16). Two branches appeared on either side of the DDFT (Figure 5F23), which was the SDFT (Figure 5F24), and then shifted position to lie deep within the DDFT. The ligaments of the fetlock joint were recognizable and well-defined, exhibiting various intermediate signal intensities. The easily identifiable ligaments in this joint included the straight, collateral sesamoidean, and axial and abaxial collateral ligaments (Figures 5F20,21,22,G25,26).

At the extremities of the proximal and middle phalanges (Figures 5G6,8), a high signal intensity was observed in the cancellous bone. In contrast, the cortical bone exhibited a noticeably low signal intensity, while the medullary cavities of the proximal and middle phalanges displayed high signal intensity. A thin plate with high signal intensity allows for the clear separation of the articular cartilage of the second phalanx (Figure 5G13) from the surrounding bone features. The ligaments of the pastern joint were well-defined and demonstrated intermediate signal intensity. The detected ligaments included the collateral ligaments of the pastern joint (Figures 5G27,28). The digital cushion showed heterogeneous high signal intensity (Figure 5F14).

### 3.3 US of the pes region

**Figure 6:**
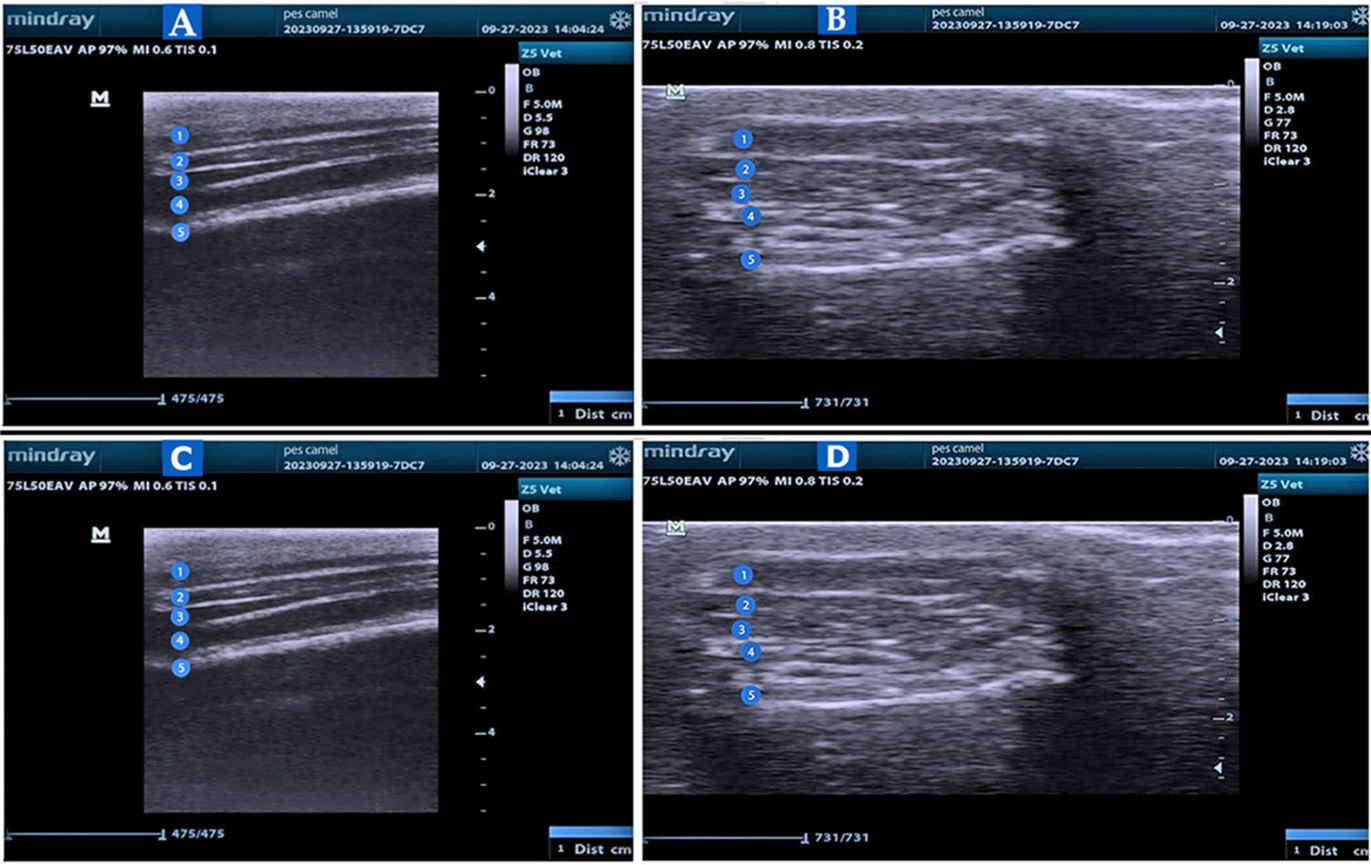
US of the pes region of the *Camelus dromedaries*. Longitudinal US images to show the plantar aspect of the large metatarsal bone in the pes region (Views **A** and **B**) to illustrate: 1. Skin; 2. Tendon of the superficial digital flexor muscle; 3. The tendon of the deep digital flexor muscle; 4. Suspensory ligament; and 5. Bone surface. Transverse US images to show the dorsal view (View **C**) and plantar view (View **D**) at the level of the metatarsophalangeal joint to show the following: 1. common digital extensor tendon or long digital extensor tendon; 2. metatarsal; 3. proximal phalanx; 4. fetlock joint; 5. axial proximal sesamoid bone; and 6. abaxial proximal sesamoid bone.

The echogenicity of the SDFT varied from medium to slightly hypoechoic. The SDFT exhibited a lengthy linear fiber pattern in the longitudinal images throughout its entire length, resembling densely packed, rough, echogenic structures that ultimately appeared as spreading linear formations (Figure 6A2). The paratendon displayed a hyper-attenuated appearance, easily distinguishable from the tendon fibers due to its higher echogenicity. On both sides of the transverse images at all levels, the SDFT appeared hypo- to medium-echogenic, with fibers visible as hyperechoic spots that were roughly uniformly distributed throughout the tendon’s hypoechoic matrix (Figure 6B2).

At all imaging levels, the echogenicity of the DDFT was higher than that of the SDFT (Figure 6A3). When distinguishing between the DDFT proximally and the SDFT distally, the SDFT paratendon appeared hyperechoic. Longitudinal images displayed the tendon fibers as roughly arranged, hyperechoic, parallel lines. The DDFT was observed to be round and well-defined in the transverse images at all levels, with pinpoint white echoes indicating the tendon fibers (Figure 6B3). Both the SDFT and DDFT were less echogenic than the SL. The longitudinal images illustrated a parallel fiber pattern with white echoes, showing the SL as a homogeneous, echogenic structure (Figure 6A4). The fiber pattern of the SL appeared as evenly spaced pinpoint white echoes in the transverse image (Figure 6B4).

In all images of the plantar views of the metatarsal, the bone surface appeared as hyperechoic reflections beneath the suspensory ligament (Figures 6A,B5). The transverse image of the fetlock joint was examined using US from a dorsal position while in an extended posture. The joint cavities of the metatarsophalangeal joint were identified via the dorsal aspect (Figure 6C4). They were noticeable as an anechoic gap on the hyper-echogenic bone surface of the metatarsal and proximal phalanx (Figures 6C2,3), with no detectable synovial fluid present. Additionally, the common digital extensor tendon and long digital extensor tendon were observed at the dorsal aspect of the fetlock joint as hyperechoic bands (Figure 61). The proximal sesamoid bones were observed on the plantar side (Figures 6D1,2).

## 4 Discussion

Our investigation is the first to use the 3D reconstruction render volume CT (3D-VR-CT) imaging technique for the pes region of *Camelus dromedarius*, which includes metatarsal bones. This approach marks a departure from previously published anatomical analyses that primarily focused on camel digits, which only employed X-ray and CT methods (20, 23). Our study utilized various imaging techniques, including CT, MRI, and US, to describe the anatomical features of the pes region and diagnose issu*es* within it. We focused particularly on 3DVR-CT. Our study is significant, given that digit and metatarsal problems are prevalent in ruminants—particularly camels. Recognizing abnormalities in these structures requires a thorough understanding of their normal anatomy. The complex structure of the digital region, which consists of joints, ligaments, and tendons, has been described in ruminants such as one-humped camels (24) and bovine digits (11). For the metatarsals, 3D reconstruction CT and MRI imaging were used for the first time. Nonetheless, several earlier studies (20, 25) focused solely on the camel’s digits, whereas Nomir et al. (11) studied the pes area of zebu cattle.

Our findings reveal that the incomplete fusion between the third and fourth metatarsal bones of *Camelus dromedarius* led to the formation of the metatarsal skeleton, as their proximal four-fifth portion contained an internal vertical bony septum that extended along the fusion line of the two bones, completely dividing the medullary cavity of the proximal extremity of the fused bones. However, this septum was absent in the distal one-fifth portion, similar to what was described in *Camelus dromedarius* (20). The fusion of the two large metatarsal bones, except for their distal portions, is also noted in bovine species (11), and similar fusions were observed in the large metacarpal bones of the Manus region in one-humped camels (26). However, the Egyptian Baladi bull features a short crest, while the Zebu bull has an extensive crest that is distal from the proximal extremity (21). Our findings indicate that the distal extremity of the fused large metatarsal bones is divided into two parts by the sagittal intercondyloid groove, forming the medial and lateral trochlea, as reported in one-humped camels (5), in the chital (27), and in the bovine (11, 21). We also found that the V-shaped intertrochlear notch is similar to those described in one-humped camels (5), appearing inverted and V-shaped with two trochleae. Our researchers observed the absence of small metatarsal bones, which is consistent with findings in one-humped camels (5). In contrast, zebu cattle have a small second metatarsal bone on the medioplantar surface of the large fourth metatarsal bone (11).

The application of 3DVR-CT imaging on the pes region of *Camelus dromedarius* allowed for a detailed description of its bony structure from all angles. It also provided an opportunity to study the articular surfaces, as demonstrated in the pes region of zebu cattle (11). Radiologists can use the 3D reformatted imaging technique to evaluate related disorders and better explain the architecture of the temporal bone by identifying and highlighting individual skeletal and soft-tissue aspects of different anatomical structures (28). Anatomical models may be created using 3D printing technologies based on 3D imaging, which supports education, enhances understanding, facilitates research, and improves diagnosis (24, 29).

The current CT findings indicate that the extensor tendons appear as a narrow, undifferentiated transverse band on the dorsal aspect of the fused third and fourth metatarsal bones, with indistinct margins for each tendon. On the plantar aspect, the deep and SDFTs are located adjacent to the interosseous muscle. In our study, we observed that the tendon of the extensor digitorum longus in the pes region divides at the distal crural region into a larger lateral tendon that bifurcates above the fetlock, forming the common digital extensor tendons, and a smaller medial component that becomes the proper extensor of the medial digit. The proper extensors produce a distoplantar-directed limb that is inserted abaxially on the proximal aspect of the middle phalanx. Our findings contradict the study by Smuts and Bezuidenhout (30), which suggested that the proper extensors of the long digital extensor tendon are attached abaxially on the distal aspect of the proximal phalanx.

The common extensor tendon, mapped along its distal course on the lateral border in this analysis, is facially related to the *extensor digitorum lateralis* tendon in the pes region and inserts at the proximal end of the middle phalanx. Smuts and Bezuidenhout (30) found that the lateral digital extensor tendon enters the distal end of the proximal phalanx; however, there is some dispute regarding these findings. In contrast, in cattle, the lateral digital extensor tendon crosses the lateral surface of the hock and terminates on the dorsal side of the second phalanx of the lateral digit (31).

In our investigation, the short digital extensor muscle, referred to as *M. extensor digitorum brevis*, is located in the pes region beneath the tarsus, at the angle between the long extensor muscle and *M. extensor digitorum lateralis*. This muscle emerges from the fibrous mass across the dorsal tarsal side and attaches to the lateral surface of the long extensor muscle. These findings are widely supported by Smuts and Bezuidenhout (30). However, in cattle, the tendon of the long extensor serves as the insertion point for the short digital extensor (M. extensor digitalis brevis), making it comparable to that in horses, as noted by Getty (31).

The current findings reveal that when the proximal and middle phalanges and fused metatarsal bones are viewed dorsally, the adjacent extensor tendons appear as a narrow strap on the CT transverse images. El-Shafey et al. (26) determined that while the flexor tendons on the fused large metatarsal (third and fourth) bones and the proximal and middle phalanges resemble a roughly rounded mass, their distinct definition is indistinguishable due to a physical variation in density of less than 0.5% in both camels and buffalo (26). Our CT images indicate that the interdigital ligament corresponds with those demonstrated in one-humped camels (26) and attaches the third and fourth digits from the proximal interphalangeal joint to the coffin joint. In contrast, two interdigital ligaments in buffalo are recognized as the proximal and distal interdigital ligaments. The proximal ligament connects the third and fourth digits along their interdigital surfaces, while the distal ligament connects these digits at the distal interphalangeal joints (26). An incomplete hyperattenuated vertical and “wedge” septum, indicating the incomplete fusion of the proximal and distal ends of these bones, is observed in the present coronal and transverse CT views between the III and IV metatarsal bones (11).

The current findings reveal that at the distal extremity of the fused third and fourth metatarsal bones, the *interosseous medius muscle* (suspensory ligament) appears as a hyperdense structure. It is clearly visualized as four oval structures representing both the axial and abaxial parts, in addition to the deep and SDFTs. Additionally, the *interosseous medius muscle* (suspensory ligament) is distinctly observed as four oval structures on the plantar aspect of the large metatarsal bone, depicted by the axial and abaxial sections. The four oval structures of the *interosseous medius muscle*, as observed using MRI imaging techniques, are consistent with the findings of Ibrahim et al. (32) and Ibrahim et al. (33). Our findings indicate a more echogenic suspensory ligament compared to the superficial and deep digital flexor tendons, influenced by the animal’s working ability and environmental conditions. These results align with the work of Abedellaah et al. (34), which demonstrated that in both longitudinal and transverse views, suspensory ligament fibers consistently exhibit pinpoint white echoes. In contrast, these observations are inconsistent with the findings described by Soroori et al. (35), who noted that the DDFT shows the highest echogenic properties at all levels. The form and echogenicity of the SDFT, DDFT, and SL differ in *Camelus dromedarius* between the phalangeal region and the proximal, middle, and distal thirds of the cannon bone; however, there is no difference between living animals and cadaveric specimens (35). In horses, the flexor tendons, accessory ligament, third metatarsal bone, and second and fourth metatarsal bones are connected to the tendinous suspensory ligament in that order (36).

Our application of the MRI imaging technique to the ligaments of the coffin and pastern joints reveals that the palmar and plantar ligaments of the pastern joint, as well as the ligaments of the navicular cartilage, may not have differentiated from the ligaments that comprise the collateral ligaments of the pastern joint and the interdigital, collateral, and dorsal ligaments of the coffin joint. These findings concur with those of Ibrahim et al. (33). Nevertheless, according to El-Shafey and Abd Al-Galil (25), the ligaments in these joints were not observed in earlier research on camels (25). Due to their wide joints, strong tendons and ligaments, digital cushions, footpads, and capsules that restrict physiological movement, camels exhibit fewer degenerative changes in their interphalangeal joints compared to other domestic animals (40).

In the current study, thin intermediate signal intensities were observed along the margins of low signal intensities found in the joint capsules of the fetlock, pastern, and coffin joints. These results concur with those reported in camels (32, 33) and cattle (37). Similar to the findings in camels (25, 32), the current MRI images show difficulty in visualizing the cruciate and short sesamoidean ligaments. According to our MRI findings, the DDFT appears in the images as an “oval” structure with low signal intensity and distinct outlines, which is consistent with earlier research in camels (25, 32). The SDFT in the MRI images shows a rounded structure with low signal intensity. While Nahas et al. (16) used a comprehensive protocol with T1, T2, PD, and STIR sequences, the current study and previous research by El-Shafey and Abd Al-Galil (25) and Ibrahim et al. (32) utilized MRI with a single T1-weighted sequence to examine camel distal limbs. The current US findings indicate that the DDFT was more echogenic than the SDFT at all imaging levels. The DDFT was distinguished from the SDFT proximally and from the suspensory ligament (SL) distally by the hyperechoic appearance of the SDFT paratendon.

Our investigation found that *Manica flexoria* was not detected in the CT or MRI images of the pes regions, which is consistent with previous studies by El-Shafey and Kassab (20), El-Shafey et al. (26), and El-Shafey and Abd Al-Galil (25). However, Ibrahim et al. (33) provided a comprehensive illustration of *Manica flexoria* in the pes region of camels using gross anatomical sections, CT, and MR images. Conversely, El-Shafey and Kassab (20), El-Shafey et al. (26), and El-Shafey and Abd Al-Galil (25) reported that the CT imaging method revealed the existence of *Manica flexoria* in the buffalo’s pes region, as well as in horses (38).

According to our analysis, the nails exhibit high signal intensity, while the sole appears as a layer of low signal intensity. The digital cushion demonstrates heterogeneous high signal intensity in the MRI images. These findings are consistent with those of Ibrahim et al. (33) and El-Shafey and Abd Al-Galil (25). Our application of CT and MRI imaging techniques revealed that the distal sesamoid bone lacks any bony or cartilaginous structures, whereas Ibrahim et al. (33) and El-Shafey and Abd Al-Galil (25) described the distal sesamoid as navicular cartilage using MRI imaging. Concurrently, the proximal, middle, and distal phalanges merge to form the bovine digit, along with the proximal and distal sesamoid bones (39). Our findings indicate that the digital cushions help maintain low pressure, reduce tension, and absorb shocks. In this study, camel CT scans reveal the presence of digital cushions, which aligns with the findings of Smuts and Bezuidenhout (30), El-Shafey et al. (26), and El-Shafey and Kassab (20).

## 5 Conclusion

Our investigations utilized CT, MRI, and US to provide detailed anatomical information on the pes region of *Camelus dromedarius*, with a particular focus on 3DVR-CT. Our results demonstrated the feasibility and efficacy of these imaging modalities for precise visualization of this region. CT, MRI, and US, along with 3DVR-CT, can now be used to evaluate anatomical structures in *Camelus dromedarius*, thereby improving the diagnosis and treatment of various conditions and facilitating the interpretation of specific clinical diseases in the pes region.

## Data Availability

The original contributions presented in the study are included in the article/supplementary material, further inquiries can be directed to the corresponding authors.

## References

[1] Adah AS Ayo JO Adah DA. Unique physiological and behavioural adaptive features of the one-humped camel (*Camelus dromedarius*) to arid environments. J App Vet Sci. (2023) 8:57–64. doi: 10.21608/javs.2022.168375.1184

[2] Roshdy K Massoud D Al-Otaibi AM Abumandour MMA. Histology, histochemistry and fine structure of the lacrimal gland in the one-humped camel (*Camelus dromedarius*). Anat Histol Embryol. (2024) 53:e13051. doi: 10.1111/ahe.13051, PMID: 38741549

[3] Roshdy K Morsy K Abumandour MM. Microscopic focus on ependymal cells of the spinal cord of the one-humped camel (*Camelus dromedarius*): histological, immunohistochemical, and transmission microscopic study. Microsc Res Tech. (2021) 85:1238–47. doi: 10.1002/jemt.23990, PMID: 34817902

[4] FAO (2016). Food and agriculture organization. Available online at: http://www.fao.org/faostat/en/#home (Accessed June 20, 2016).

[5] Hifny A Misk NA Semieka MA. Radiographic studies on the Manus and pes of camel and cattle. J Camel Prac Res. (1995) 1:87–91.

[6] Liebich H-G König HE. Veterinary anatomy of domestic mammals: textbook and colour atlas. 7th edition. New York, United States: Thieme (2020).

[7] Yousef AT Hussein MK Hamed MA Farrag FA Abumandour MMA Hamoda H. Morphological and radiographic studies on the Manus region in the Arabian one-humped camel (Camelus dromedaries). Anat Histol Embryol. (2024) 53:e13040. doi: 10.1111/ahe.13040, PMID: 38623947

[8] Solano L Barkema HW Pajor EA Mason S Leblanc SJ Heyerhoff JZ. Prevalence of lameness and associated risk factors in Canadian Holstein-Friesian cows housed in freestall barns. J Dairy Sci. (2015) 98:6978–91. doi: 10.3168/jds.2015-9652, PMID: 26254526

[9] Gahlot T.K. “Lameness in camels.” In "*Proceedings of the International Camel Conference" Recent trends in Camelids research and Future strategies for saving Camels", Rajasthan, India, 16–17 February 2007." College of Veterinary & Animal Science*, (2007), 2007.

[10] Abumandour MMA El-Bakary R Enany ES Karkoura A Farid S. Biological aspects of the nasal turbinates of the Anatolian shepherd dog captured from Egypt: using computed tomography, histological, and scanning electron microscopic observations. Microsc Res Tech. (2022) 85:927–39. doi: 10.1002/jemt.23962 34651363

[11] Nomir AG El Sharaby A Abumandour MMA. Anatomical studies on the PES region of zebu cattle (*Bos Taurus indicus*) with special references to 3D computed tomography imaging technique. BMC Vet Res. (2024) 20:1–18. doi: 10.1186/s12917-024-03940-0 38459515 PMC10921674

[12] Abumandour MM Bassuoni NF El-Gendy S Karkoura A El-Bakary R. Cross-anatomical, radiographic and computed tomographic study of the stifle joint of donkeys (*Equus africanus asinus*). Anat Histol Embryol. (2020) 49:402–16. doi: 10.1111/ahe.12543, PMID: 32175631

[13] Haddad SS Abdel-Megeed N Abumandour MMA. Prenatal and postnatal development of New Zealand white rabbit (*Oryctolagus cuniculus*) teeth: histological and computed tomography aspects. Zoomorphology. (2024) doi: 10.1007/s00435-024-00649-4

[14] Ibrahim A Rashwan A El Sharaby A Abumandour M Nomir A. Thoracic cavity of the Shirazi cats: new insights using computed tomography and magnetic resonance imaging. Anat Histol Embryol. (2024) 53:e13005. doi: 10.1111/ahe.13005 38018270

[15] Calhoun PS Kuszyk BS Heath DG Carley JC Fishman EK. Three-dimensional volume rendering of spiral CT data: theory and method. Radiographics. (1999) 19:745–64. 10336201 10.1148/radiographics.19.3.g99ma14745

[16] Nahas AE Almohamad Z Hagag U. Ultrasonography, computed tomography and magnetic resonance imaging of the dromedary camel distal limbs. BMC Vet Res. (2024) 20:12. doi: 10.1186/s12917-023-03855-2, PMID: 38183041 PMC10768528

[17] Sherlock C Mair T Ireland J Blunden T. Do low field magnetic resonance imaging abnormalities correlate with macroscopical and histological changes within the equine deep digital flexor tendon? Res Vet Sci. (2015) 98:92–7. doi: 10.1016/j.rvsc.2014.12.008, PMID: 25555604

[18] Tamura S Tamura Y Tsuka T Uchida K. Sequential magnetic resonance imaging of an intracranial hematoma in a dog. Vet Radiol Ultrasound. (2006) 47:142–4. doi: 10.1111/j.1740-8261.2006.00120.x, PMID: 16553145

[19] Kofler J Hittmair K. Diagnostic ultrasonography in animals - continuation of the clinical examination? Vet J. (2006) 171:393–5. doi: 10.1016/j.tvjl.2005.02.004, PMID: 16624705

[20] El-Shafey A Kassab A. Computed tomography and cross-sectional anatomy of the metatarsus and digits of the one-humped camel (*Camelus dromedarius*) and Buffalo (*Bos bubalis*). Anat Histol Embryol. (2013) 42:130–7. doi: 10.1111/j.1439-0264.2012.01174.x 22776073

[21] El Sharaby AA Abd El-Fatah MA Abumandour MMA Nomir AG. Morphological comparison between the large metatarsal bone of zebu bull (*Bos Taurus indicus*) and Egyptian Baladi bull (*Bos Taurus Taurus*): new insights by gross, morphometric, and computed tomography. J Morpholog Sci. (2021) 38:291–8. doi: 10.51929/jms.38.50.2021

[22] Nomina Anatomica Veterinaria, N. International committee on veterinary gross anatomical nomenclature and authorized by the general assembly of the world association of veterinary anatomist. 6th ed. Knoxville: Ghent. Editorial Committee Hanover (Germany), Ghent (Belgium), Sapporo (Japan), Columbia, MO (U.S.A.), Rio de Janeiro (Brazil). (2017).

[23] Badawy AM Marzok MA Eshra EA. Computed tomographic arthrography of the normal dromedary camel carpus. Vet Comp Orthop Traumatol. (2016) 29:188–94. doi: 10.3415/VCOT-15-06-0112, PMID: 26898661

[24] Al Aiyan A King FC Aldarwich A Kishore U Shawaf T. Arthrocentesis approaches to the phalangeal joints of the one humped camel (*Camelus dromedarius*). Sci Rep. (2023) 133:17354. doi: 10.1038/s41598-023-44391-1 PMC10576090 37833397

[25] El-Shafey AA Abd Al-Galil ASA. Magnetic resonance imaging of the one-humped camel (*Camelus dromedarius*) digits. J Am Sci. (2012) 8:549–56.

[26] El-Shafey A Sayed-Ahmed A El-Shafey A Sayed-Ahmed A. Computed tomography and cross sectional anatomy of the metacarpus and digits of the one-humped camel and Egyptian water buffalo. Int J Morphol. (2012) 30:473–82.

[27] Yadav SC Joshi S Mathur R Choudhary OP. Gross and biometrical studies on pelvic bones of chital (*Axis axis*). Indian J Vet Anatomy. (2012) 24:87–8.

[28] Choplin RH Buckwalter KA Rydberg J Farber JM. CT with 3D rendering of the tendons of the foot and ankle: technique, normal anatomy, and disease. Radiographics. (2004) 24:343–56. doi: 10.1148/rg.242035131 15026585

[29] Choudhary OP. Three-dimensional computed tomography reconstructions: a tool for veterinary anatomy education. Ann Med Surg (Lond). (2021) 67:102497. doi: 10.1016/j.amsu.2021.102497 34295462 PMC8282458

[30] Smuts MMS Bezuidenhout AJ. Anatomy of the dromedary. USA: Oxford University Press (1987).

[31] Getty R. The anatomy of the domestic animals, vol. 1. 5th ed. Philadelpshia, USA: W.B. Saunders Company (1975).

[32] Ibrahim AH Adam ZE Tawfiek MG. Normal cross-sectional anatomy and magnetic resonance imaging of pastern and coffin joints in camel. J Vet Med Res. (2019) 26:271–9. doi: 10.21608/jvmr.2019.67970

[33] Ibrahim AH Adam ZE Tawfiek MG. Cross-sectional anatomy, magnetic resonance imaging and computed tomography of fetlock joint in camel. J Vet Med Res. (2019) 26:258–70. doi: 10.21608/jvmr.2019.67969

[34] Abedellaah BA Elrashidy MH Rashed R. Ultrasonographic study of the flexor tendons and suspensory ligament of the metacarpal/metatarsal region in one-humped camel (Camelus dromedaries). Indian J Vet Surg. (2014) 35:127–30. doi: 10.1016/j.bjbas.2017.03.003

[35] Soroori S Masoudifard M Vajhi AR Rostami A Salimi M. Ultrasonography study of tendons and ligaments of metacarpal region in the camel (*Camelus dromedarius*). Int J Vet Res. (2011) 5:85–8.

[36] Gadallah SM Sharshar AM Elsharkawy SA Fadel MS. Ultrasonographic description of tendons and ligaments at the palmar (plantar) aspect of the distal limb in the one humped camel (*Camelus dromedarius*). Aust Vet J. (2023) 101:397–408. doi: 10.1111/avj.13268, PMID: 37544650

[37] Hagag U Tawfiek M. Ultrasonography, computed tomography and magnetic resonance imaging of the bovine metacarpo/metatarsophalangeal joint. Vet J. (2018) 233:66–75. doi: 10.1016/j.tvjl.2018.01.001, PMID: 29486882

[38] Vanderperren K Ghaye B Snaps FR Saunders JH. Evaluation of computed tomographic anatomy of the equine metacarpophalangeal joint. Am J Vet Res. (2008) 69:631–8. doi: 10.2460/ajvr.69.5.631, PMID: 18447794

[39] Dyce KM Sack WO Wensing CJG. Text book of veterinary anatomy. 5th ed. Philadelphia, London and Toronto: W.B. Saunders Company (2010).

[40] Lotfia SF Hegazy AA Sheta EM Waly Y. An approach to the phalangeal region affections in camel (Camelus dromedarius). Veterinary Medical Journal Giza (1996) 44:259–272.

